# Intravenous Administration of Pyroglutamyl Apelin-13 Alleviates Murine Inflammatory Pain via the Kappa Opioid Receptor

**DOI:** 10.3389/fnins.2020.00929

**Published:** 2020-09-08

**Authors:** Shuangyu Lv, Xiaomei Zhang, Yu Feng, Yuchen Zhou, Binbin Cui, Yanjie Yang, Xinchun Wang

**Affiliations:** ^1^Institute of Molecular Medicine, School of Basic Medical Sciences, Henan University, Kaifeng, China; ^2^Key Laboratory of Clinical Resources Translation, The First Affiliated Hospital of Henan University, Kaifeng, China

**Keywords:** (pyr)apelin-13, inflammatory pain, opioid receptor, dynorphin, prefrontal cortex

## Abstract

Apelin is an endogenous neuropeptide, which has wide distribution in central nervous system and peripheral tissues. Pyroglutamyl apelin-13 [(pyr)apelin-13] is the major apelin isoform in human plasma. However, the role of peripheral (pyr)apelin-13 in pain regulation is unknown. The aim of this study was to investigate the effect of the peripheral injection of (pyr)apelin-13 on inflammatory pain using the formalin test as well as to evaluate the mechanistic basis for the effect. Results showed intravenous (i.v.) injection of (pyr)apelin-13 (10, 20 mg/kg) to significantly decrease licking/biting time during the second phase of the mouse formalin test. In contrast, i.v. injection of apelin-13 had no influence on such effect. Intramuscular injection of (pyr)apelin-13 reduced licking/biting time during the second phase only at a dose of 20 mg/kg. The antinociception of i.v. (pyr)apelin-13 was antagonized by the apelin receptor (APJ, angiotensin II receptor-like 1) antagonist, apelin-13(F13A). (pyr)apelin-13 (i.v. 20 mg/kg) markedly upregulated *Aplnr* and *Adcy2* gene expression in the prefrontal cortex, whereas *Fos* gene expression was downregulated. The antinociception of i.v. (pyr)apelin-13 was blocked by the opioid receptor antagonist naloxone and the specific kappa opioid receptor (KOR) antagonist nor-binaltorphimine (nor-BNI). (pyr)Apelin-13 upregulated the dynorphin and KOR gene expression and protein levels in the mouse prefrontal cortex, not in striatum. (pyr)Apelin-13 did not influence the motor behavior. Our results demonstrate that i.v. injection of (pyr)apelin-13 induces antinociception via the KOR in the inflammatory pain mouse model.

## Introduction

Apelin, also known as APLN, is the endogenous ligand for the apelin receptor (APJ, angiotensin II receptor-like 1), an orphan G protein-coupled receptor (GPCR) (Tatemoto et al., 1998). APJ, also known as APLNR, was originally identified by O’Dowd et al. (1993). The APJ receptor is composed of 380 amino acid residues, the sequence of which has considerable similarity to that of the angiotensin II type 1 (AT1) receptor (54% for the transmembrane domains and 30% for the entire sequence). Nevertheless, angiotensin II does not bind to APJ (O’Dowd et al., 1993). Apelin is an endogenous peptide, which was first isolated from bovine stomach extracts (Tatemoto et al., 1998). The preproprotein of apelin (known as preproapelin) consists of 77 amino acids, coded by *APLN* which is located on chromosome Xq25–26.1 (Lee et al., 2000). When preproapelin was enzymatically hydrolyzed, several bioactive fragments are produced, including apelin-36, apelin-19, apelin-17, apelin-13, and apelin-12 (Habata et al., 1999; Hosoya et al., 2000). It is notable that (pyr)apelin-13, the pyroglutamate modified form of apelin-13, was identified as the major apelin isoform in human plasma (Zhen et al., 2013). (pyr)Apelin-13 was shown to be more structurally stable than apelin-13 (Hosoya et al., 2000) in that the N-terminal pyr protects from exopeptidase degradation (Masri et al., 2005). A clinical study demonstrated that (pyr)apelin-13 was to be the predominant cardiac isoform in patients with coronary artery disease (Maguire et al., 2009).

The apelin/APJ system is involved in multiple physiological and pathophysiological functions, including cardiovascular regulation, cancer (Yang et al., 2016; Patel et al., 2017), ischemia/reperfusion injury (Yang et al., 2015), fluid homeostasis, angiogenesis (O’Carroll et al., 2013), and liver metabolism (Lv et al., 2017). Recently, a role for the apelin/APJ system in neurological disease and mental disorders has attracted considerable attention (Lv S.-Y. et al., 2020). Intracerebroventricular (i.c.v.) injection of apelin attenuated depressive-like behavior in rats with chronic stress (Dai et al., 2018; Zhang et al., 2019). Apelin-13 (i.c.v.) alleviated memory performance deficits in a rat model of chronic water-immersion restraint stress (Shen et al., 2019) and cognitive deficits in a streptozotocin-induced rat model of Alzheimer’s disease (Luo et al., 2019), each of which was mediated by brain-derived neurotrophic factor (BDNF) signaling. Fan et al. (2017, 2018) found that intraperitoneal (i.p.) injection of apelin-13 significantly mitigates mouse anxiety-like behavior induced by chronic normobaric hypoxia by inhibition of the nuclear factor kappa-B (NF-κB) pathway. In addition, apelin had anti-neuroinflammatory (Zhou et al., 2019), anti-apoptotic, and neuroprotective effects (Liu et al., 2019). Further, apelin played a role in Parkinson’s disease (Acar et al., 2019) and post-traumatic stress disorder (Chen et al., 2019).

Apelin/APJ genes were ubiquitously expressed in various organs of humans and rodents, with the highest expression in lung, heart, adipose tissue, brain, gastrointestinal tract, liver, kidney, and the cardiovascular system (Habata et al., 1999; Kawamata et al., 2001; Medhurst et al., 2003). Apelin and APJ had been detected in the amygdala, hypothalamus, dorsal raphe nucleus (DRN), and the spinal cord (Hosoya et al., 2000; O’Carroll et al., 2000; Reaux et al., 2001), which were anatomic sites of descending pain modulation pathways. Our previous report showed that i.c.v. and intrathecal (i.t.) administration of apelin-13 induced an antinociceptive effect in an acetic acid–induced mouse visceral pain model (Lv et al., 2012). However, a role for peripheral (pyr)apelin-13 in inflammatory pain is unknown.

The formalin-test pain model is more relevant to clinical pain than other models in that the pain response is evoked by a continuous and acute stimulus that is reproducible and quantifiable (Reeta et al., 2006). This model is used to assess potential antinociceptive agents, and it has two pain phases, a first (early) phase and a second (late) phase. The first phase is initiated by direct stimulation of nociceptors and the second phase develops in parallel with inflammatory processes (Dubuisson and Dennis, 1977). The present study was designed to assess the effect of intravenous (i.v.) injection and intramuscular (i.m.) injection of (pyr)apelin-13 on the inflammatory pain induced by the formalin test. The mechanistic basis for the underlying effect was explored by quantitative real-time polymerase chain reaction (RT-qPCR), western blot, and ELISA.

## Materials and Methods

### Animals

Male Kunming mice (6–8 weeks of age, 20 ± 1 g) were supplied by the Animal Center of Henan Province (Zhengzhou, China). The animals were kept in standard cages (5–6 mice/cage) in a room at 22 ± 1°C, with a 12 h/12 h light/dark cycle, 50–60% relative humidity, free access to water, and standard food. The mice were allowed to adapt to this environment for at least 7 days before experiments. This study and the animal experimental protocols were performed in accordance with the Committee of Medical Ethics and Welfare for Experimental Animals, Henan University School of Medicine (No. HUSOM2016-042).

### Chemicals and Drug Administration

The peptides, apelin-13, (pyr)apelin-13, and apelin-13(F13A) were obtained from GL Biochem (Shanghai) Ltd. (Shanghai, China). The amino acid sequence of (pyr)apelin-13 was “Pyr-Arg-Pro-Arg-Leu-Ser-His-Lys-Gly-Pro-Met-Pro-Phe,” and the amino acid sequence of apelin-13 was “Gln-Arg-Pro-Arg-Leu-Ser-His-Lys-Gly-Pro-Met-Pro-Phe.” Apelin-13(F13A) was the APJ receptor antagonist. Morphine hydrochloride was supplied by The First Affiliated Hospital of Henan University (Kaifeng, China). Naloxone (opioid receptor antagonist) and nor-binaltorphimine dihydrochloride (nor-BNI, KOR antagonist) were purchased from Sigma-Aldrich (St. Louis, MO, United States). The chemicals were dissolved in sterile saline and stored at -20°C until the time of injection. The drugs were i.v. injected into mouse tail veins at a constant rate of 0.01 ml per second or were intramuscularly (i.m.) administered into buttock sites in a volume of 0.1 ml.

### Formalin Test

A model of acute inflammatory pain was established using the formalin test. The test was performed as described previously (Tjolsen et al., 1992; Lv et al., 2013). Briefly, the animals were placed into a transparent glass cylinder (height, 20 cm; diameter, 15 cm) individually, with a mirror angled at 45° below the surface of the cylinder that allowed free viewing of nociceptive-related behaviors. For adaptation the mice were placed in the glass cylinder 30 min before testing. After habituation, a 1.0% formalin solution was intraplantar (i.pl.) injected into the dorsal surface of the right hind paw in a volume of 20 μl/mouse. The amount of time the animal spent licking or biting the injected paw was recorded for 30 min using a stopwatch. The time period from 0 to 10 min (phase 1) represents the acute pain phase (early phase) and the time period from 10 to 30 min (phase 2) represents the inflammatory pain phase (late phase). The observers (X.Z. and Y.F.) were blind to the study protocol.

In formalin test, the experiment consisted of four sections: (1) To evaluate the analgesic effects of peripheral (pyr)apelin-13, (pyr)apelin-13 were administered by i.v. 30 min before formalin treatment. Morphine served as a positive control. In this section, animals were divided into five groups: saline (*n* = 10), 2 mg/kg (pyr)apelin-13 (*n* = 9), 10 mg/kg (pyr)apelin-13 (*n* = 8), 20 mg/kg (pyr)apelin-13 (*n* = 8), and 2 mg/kg morphine (*n* = 9). (2) To examine the analgesic effects of peripheral (pyr)apelin-13, mice were divided four groups: saline (*n* = 10) and apelin-13 (2, 10, and 20 mg/kg; *n* = 8, 7, and 8, respectively). The drugs were i.v. injected 30 min before formalin treatment. (3) To examine the analgesic effects of i.m. (pyr)apelin-13, mice were divided four groups: saline (*n* = 9), (pyr)apelin-13 (10 and 20 mg/kg; *n* = 9 and 9, respectively), and morphine (2 mg/kg, *n* = 8). The saline or (pyr)apelin-13 was i.m. injected 30 min before formalin treatment. (4) To explore the potential mechanism of the antinociception induced by (pyr)apelin-13, the animals were divided into six groups: saline (*n* = 10), 20 mg/kg (pyr)apelin-13 (*n* = 8), 20 mg/kg apelin-13(F13A) (*n* = 8), 2 mg/kg naloxone (*n* = 8), 10 mg/kg nor-BNI (*n* = 8), 20 mg/kg (pyr)apelin-13 + 20 mg/kg apelin-13(F13A) (*n* = 8), 20 mg/kg (pyr)apelin-13 + 2 mg/kg naloxone (*n* = 7), and 20 mg/kg (pyr)apelin-13 + 10 mg/kg nor-BNI (*n* = 8).

All the saline or chemicals [apelin13, (pyr)apelin-13, or antagonists] were i.v./i.m. injected 30 min before i.pl. injection of formalin. The dose of (pyr)apelin-13 was selected according to the previous reports (Chen et al., 2015; Gourdy et al., 2018). Each antagonist [apelin-13(F13A), naloxone, and nor-BNI] was mixed with (pyr)apelin-13 and then i.v. co-administered in a volume of 100 μl at one time point, respectively. This co-administrated administration method was selected following the previous reports (Lv et al., 2012, 2013), and this procedure is to minimize the total injection volume and ascertain that the compounds were similarly localized.

To investigate potential gene(s) or protein(s) involved in antinociception induced by (pyr)apelin-13, 30 min after i.v. treatment with 20 mg/kg (pyr)apelin-13 or saline, mice were i.pl. injected with formalin. Thirty minutes later, the brain tissues (prefrontal cortex, striatum, etc.) were removed, snap frozen in liquid nitrogen, and stored at -80°C until analyzed by RT-qPCR, ELISA, or western blot.

### RNA Isolation and Reverse Transcription

Frozen brain tissue was homogenized (Power Gen 125; Fisher Scientific, Pittsburgh, PA, United States) and total RNA was isolated using TRIzol Reagent (Invitrogen, Thermo Fisher Scientific, Carlsbad, CA) based on the manufacturer’s protocol. The concentration of the sample was measured with a NanoDrop 2000 UV–Vis Spectrophotometer (Thermo Fisher Scientific, Wilmington, DE, United States) and the concentration of RNA was measured by OD260/OD280. Total RNA was reverse transcribed to complementary DNA (cDNA) using a High Capacity cDNA Reverse Transcription kit (Applied Biosystems, Foster City, CA) following the product’s instruction. During the process, M-MLV reverse transcriptase and oligo dT (200 U/μl) were applied in a final volume of 50 μl.

### RT-qPCR

The mRNA levels were assessed for the APJ receptor (*Aplnr*), proopiomelanocortin (*Pomc*), prodynorphin (*Pdyn*), proenkephalin (*Penk*), mu opioid receptor (*Oprm1*), KOR (*Oprk1*), delta opioid receptor (*Oprd1*), adenylate cyclase 1 (*Adcy1*)–*Adcy9*, brain-derived neurotrophic factor (*Bdnf*), FBJ osteosarcoma oncogene (*Fos*), CAMP responsive element binding protein 1 (*Creb1*), down-regulator of transcription 1 (*Dr1*), early growth response protein 1 (*Egr1*), mitogen-activated protein kinase 14 (*p38α*), and signal transducer and activator of transcription 2 (*Stat2*). RT-qPCR was carried out with a 7500HT thermal cycler (Applied Biosystems) and SYBR Green master mix (Invitrogen, South San Francisco, CA, United States). The sequence of primers used for RT-qPCR assays are listed in Table 1 and were designed following previous reports. The gene for *36B4* was included as an endogenous control. After each RT-qPCR, dissociation curve analysis was conducted to ensure the specificity of the PCR amplification. The normalized expression of the target genes was calculated with the equation 2^–ΔΔCt^, and the ΔΔCt = (Ct, _Target_ - Ct, _36*B*4_)_drug_ - (Ct, _Target_ − Ct, _36B4_)_control_.

**TABLE 1:** Primer sequence used for RT-qPCR.

Primer name	Primer sequence	Size (bp)
*Aplnr*-F	5′-CCACCTGGTGAAGACTCTCTACA-3′	110
*Aplnr*-R	5′-TGACATAACTGATGCAGGTGC-3′	
*Pomc*-F	5′-AGATTCAAGAGGGAGCTGGA-3′	159
*Pomc*-R	5′-CTTCTCGGAGGTCATGAAGC-3′	
*Pdyn*-F	5′-CGGAACTCCTCTTGGGGTAT-3′	154
*Pdyn*-R	5′-TTTGGCAACGGAAAAGAATC-3′	
*Penk*-F	5′-AACAGGATGAGAGCCACTTGC-3′	474
*Penk*-R	5′-CTTCATCGGAGGGCAGAGACT-3′	
*Oprm1*-F	5′-ATCCTCTCTTCTGCCATTGGT-3′	127
*Oprm1*-R	5′-TGAAGGCGAAGATGAAGACA-3′	
*Oprd1*-F	5′-AAGTACTTGGCGCTCTGGAA-3′	125
*Oprd1*-R	5′-GCTCGTCATGTTTGGCATC-3′	
*Oprk1*-F	5′-CCGATACACGAAGATGAAGAC-3′	341
*Oprk1*-R	5′-GTGCCTCCAAGGACTATCGC-3′	
*Adcy1*-F	5′-CCGGAACATGGACCTCTACTAC-3′	284
*Adcy1*-R	5′-ATAGGTGGGAGGAGATGGACTG-3′	
*Adcy2*-F	5′-CCTGGGACCAGGTGTCATTC-3′	412
*Adcy2*-R	5′-CCTGCTTTGGGTCCCTGTAG-3′	
*Adcy3*-F	5′-TACTTCAAAAGGCAGCGCCA-3′	482
*Adcy3*-R	5′-TTGGCCAGGATCTCCCTCAG-3′	
*Adcy4*-F	5′-TTGACCCAAAGCGGGCAG-3′	248
*Adcy4*-R	5′-GCACACAGCACAGTTGTCAG-3′	
*Adcy5*-F	5′-ACTTGGCCATCTCTCTGCAC-3′	445
*Adcy5*-R	5′-TGATTCTCCGCAGCCAACTT-3′	
*Adcy6*-F	5′-GCGGTGAGGGAGAATCACTG-3′	163
*Adcy6*-R	5′-TCACACCTGTTACCTCACGC-3′	
*Adcy7*-F	5′-GCAGGTAACAGGGTCGGAG-3′	392
*Adcy7*-R	5′-AGGTCCTCAGCTCTTTGCAC-3′	
*Adcy8*-F	5′-TTGCGGAGTGGCGATAAGTT-3′	482
*Adcy8*-R	5′-ACAAAGTACTCTGGGTAGGAGC-3′	
*Adcy9*-F	5′-AAGACCAGCACCAAGGCTTC-3′	183
*Adcy9*-R	5′-GTTCTTGAACCTGAGCGGGA-3′	
*Bdnf*-F	5′-TGGCTGACACTTTTGAGCACGTC-3′	135
*Bdnf*-R	5′-GCTCCAAAGGCACTTGACTGCTGA-3′	
*Fos*- F	5′-GGTGAAGACCGTGTCAGGAGGCAG-3′	117
*Fos*-R	5′-GCCATCTTATTCCGTTCCCTTCGG-3′	
*Creb1*-F	5′-TACCCAGGGAGGAGCAATAC-3′	183
*Creb1*-R	5′-GAGGCAGCTTGAACAACAAC-3′	
*Dr1*-F	5′-TCGGCAGACATGTTGTGAGG-3′	268
*Dr1*-R	5′-TCTAGGGACACCACTCCCAG-3′	
*Egr1*-F	5′-GAGCACCTGACCACAGAGTC-3′	172
*Egr1*-R	5′-AAAGGGGTTCAGGCCACAAA-3′	
*p38α*-F	5′-CACAGGGACCTAAAGCCCAG-3′	305
*p38α*-R	5′-TTCTTCAGAAGCTCAGCCCC-3′	
*Stat2*-F	5′-GTCCTTGAACCGCTTGGAGA-3′	87
*Stat2*-R	5′-TGCGCCATTTGGACTCTTCT-3′	
*36B4*-F	5′-CGACCTGGAAGTCCAACTAC-3′	109
*36B4*-R	5′-ATCTGCTGCATCTGCTTG-3′	

*F, forward; R, reverse.*

### ELISA

Blood and prefrontal cortex were removed from mice 1 h after i.v. administration of (pyr)apelin-13 or saline. Brains were weighed, homogenized with 10% *w*/*v* phosphate buffer (0.1 M, pH 7.4), and centrifuged at 10,000 rpm for 15 min at 4°C. Serum was obtained by centrifugation of blood at 4000 rpm at 4°C for 5 min. Brain supernatants and serum were assessed for dynorphin content using the Mouse Dynorphin ELISA Kit (Shanghai Fusheng Shiye Co. Ltd., Shanghai, China) according to the manufacturer’s protocol.

### Western Blot

Protein lysates of mouse brain tissue were prepared in RIPA:PMSF (100:1) supplemented with phosphatase inhibitors. Protein concentrations were measured by the bicinchoninic acid assay (Beyotime, Shanghai, China). Samples were subjected to sodium dodecyl sulfate polyacrylamide gel electrophoresis (SDS-PAGE) and then transferred to polyvinylidene difluoride (PVDF) membranes (Pall, East Hills, NY, United States). The PVDF membranes were blocked with 5% skim milk and incubated with primary antibodies reactive with KOR (1:1000; Abcam, Cambridge, MA, United States) or beta-actin (1:1000; Beyotime), at 4°C overnight. The blots were then incubated with horseradish peroxidase (HRP)–labeled secondary antibody (Proteintech, Shanghai, China) for 1 h. The membrane was incubated with SuperSignal West Pico Chemiluminescent Substrates (Thermo Fisher Scientific, Waltham, MA, United States) and bands visualized using an automatic multifunction chemiluminescent detection system (Tanon, Shanghai, China). The signals were calculated by densitometry using ImageJ software.

### Open Field Test

The open field test was conducted in a sound-attenuated room using the universal spontaneous activity video analysis system (model no. JLBehv-LM4; Shanghai Jiliang Software Technology Co., Ltd., Shanghai, China) according to the previous report (Lv S. et al., 2020). The mouse was individually placed in the center of the apparatus, consisting of a square area surrounded by high walls (25 × 25 × 31 cm), which was equipped with a video camera above the center. The total distance traveled, velocity, and numbers of spontaneous activity were recorded. Mice were put in the apparatus for 30 min habituation after i.v. injection of saline or (pyr)apelin-13 or saline, and then immediately i.pl. injected with 1.0% formalin solution and put back in the apparatus again to test for 30 min. The apparatus was cleaned with a 10% ethanol solution after each trial. The mice were divided into four groups: saline (*n* = 11) and (pyr)apelin-13 (2 mg/kg, *n* = 10; 10 mg/kg, *n* = 9; 20 mg/kg, *n* = 10).

### Wire Hanging Test

The wire hanging test was conducted following the previous report (Niimi et al., 2009). The animal was placed on a stainless steel bar (50 cm in length, 2 mm diameter, 37 cm above the floor) at a point midway between the supports and observed for 30 s. The score was evaluated according to the following scheme: 0, fell off; 1, hung onto the wire by two forepaws; 2, hung onto the wire by two forepaws, but also attempted to climb onto the wire; 3, hung onto the wire by two forepaws plus one or both hindpaws around the wire; 4, hung onto the wire by all four paws plus tail wrapped; 5, escaped. Latency to falling off and the score were also recorded, and the cutoff latency was set at 30 s. Each mouse had three opportunities, and the inter-trial intervals were 30 min. Thirty minutes after saline or (pyr)apelin-13 treatment, formalin was i.pl. injected. After 15 min, the wire hanging test was started. The mice were divided into four groups: saline and (pyr)apelin-13 (2, 10, and 20 mg/kg, *n* = 10 per group).

### Light/Dark Aversion Test

The test was performed following De Angelis and Furlan (2000). The apparatus consisted of two compartments (27 × 21 × 14 cm), separated by a connecting gate (7 × 10 cm). One of these compartments was darkened by black paint and covered with a black cover. The other compartment was lit by a 60-W desk lamp 30 cm above. Each mouse was individually placed at the center of the bright compartment (facing away from the door). The total time spent in the light area and number of transitions between light and dark areas were measured for 5 min. Thirty minutes after saline or (pyr)apelin-13 injection, formalin was i.pl. injected. After 15 min, the light/dark aversion test was started. The mice were divided into four groups: saline (*n* = 10) and (pyr)apelin-13 (2, 10, and 20 mg/kg, *n* = 9 per group).

### Statistical Analysis

Data are presented as means ± SEM. Analysis was performed by one-way ANOVA, followed by Dunnett’s test for multiple comparisons using SPSS 16.0. The unpaired *t*-test was used to test the difference between the two groups. A *p* <0.05 was used as the criterion for statistical significance.

## Results

### The Effect of Peripheral (Pyr)Apelin-13 on the Nociceptive Response During the Mouse Formalin Test

**FIGURE 1:**
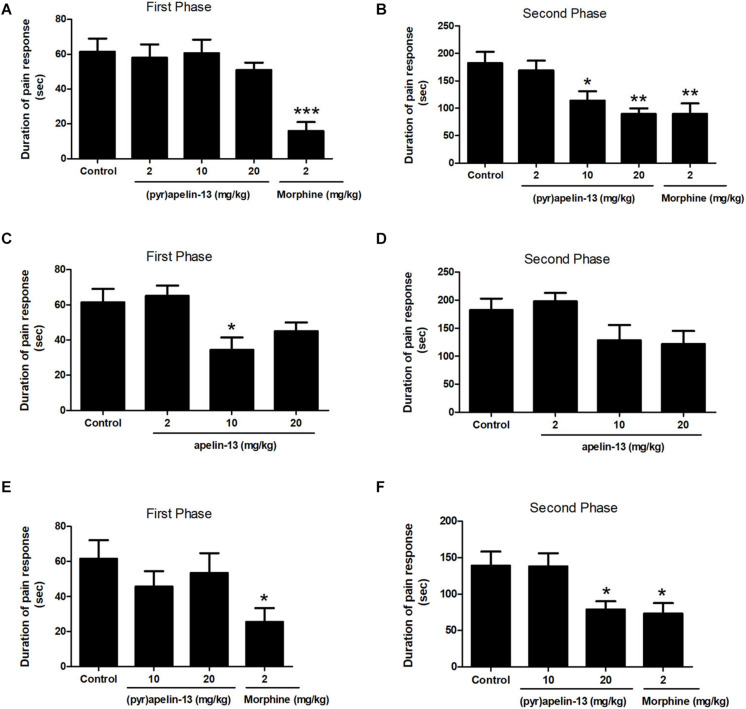
Antinociception by (pyr)apelin-13 and apelin-13 during the formalin test. **(A,B)** Normal saline (control), (pyr)apelin-13 (2, 10, 20 mg/kg), or morphine (2 mg/kg) were intravenously (i.v.) injected 30 min before intraplantar (i.pl.) injection of formalin. **(C,D)** Normal saline, apelin-13 (2, 10, 20 mg/kg), or morphine (2 mg/kg) were i.v. administered before i.pl. injection of formalin. **(E,F)** Normal saline, (pyr)apelin-13 (10, 20 mg/kg), or morphine (2 mg/kg) were i.m. administrated before i.pl. injection of formalin. Each point is the mean licking/biting time during the early phase (0–10 min) or late phase (10–30 min) of the formalin test. Data are expressed as means ± SEM. *n* = 7–10 per group. *p < 0.05, **p < 0.01, and ***p < 0.001 vs. control according to ANOVA followed by Dunnett’s test.

One-way ANOVA demonstrated i.v. administration of (pyr)apelin-13 (2, 10, and 20 mg/kg) to have no influence on licking/biting time during the first phase [*F*(3, 31) = 0.441, *p* = 0.725] (Figure 1A). However, i.v. administration of (pyr)apelin-13 produced a dose-dependent decrease in licking/biting time during the second phase [*F*(3, 31) = 6.350, *p* < 0.01] (Figure 1B). Compared with the control, (pyr)apelin-13 significantly decreased the licking/biting time at doses of 10 mg/kg (*p* < 0.05) and 20 mg/kg (*p* < 0.01).

Administration of apelin-13 (non-pyroglutamyl) markedly reduced licking/biting time during the first phase of the formalin test only at the 10 mg/kg dose (*p* < 0.05, Figure 1C) when compared with the control group. In the second phase, apelin-13 had no obvious influence on licking/biting time (2 mg/kg, *p* = 0.919; 10 mg/kg, *p* = 0.216; 20 mg/kg, *p* = 0.124; Figure 1D), compared with the control.

Injection (i.m.) of (pyr)apelin-13 (10 and 20 mg/kg) had no effect on paw licking/biting time during the first phase, compared with saline treatment (*p* = 0.452, *p* = 0.800, Figure 1E). During the second phase, i.m. administration of (pyr)apelin-13 produced a significant decrease in licking/biting time at 20 mg/kg (*p* < 0.05), not at 10 mg/kg (*p* = 0.998, Figure 1F).

### The Effect of i.v. (Pyr)Apelin-13 on APJ

**FIGURE 2:**
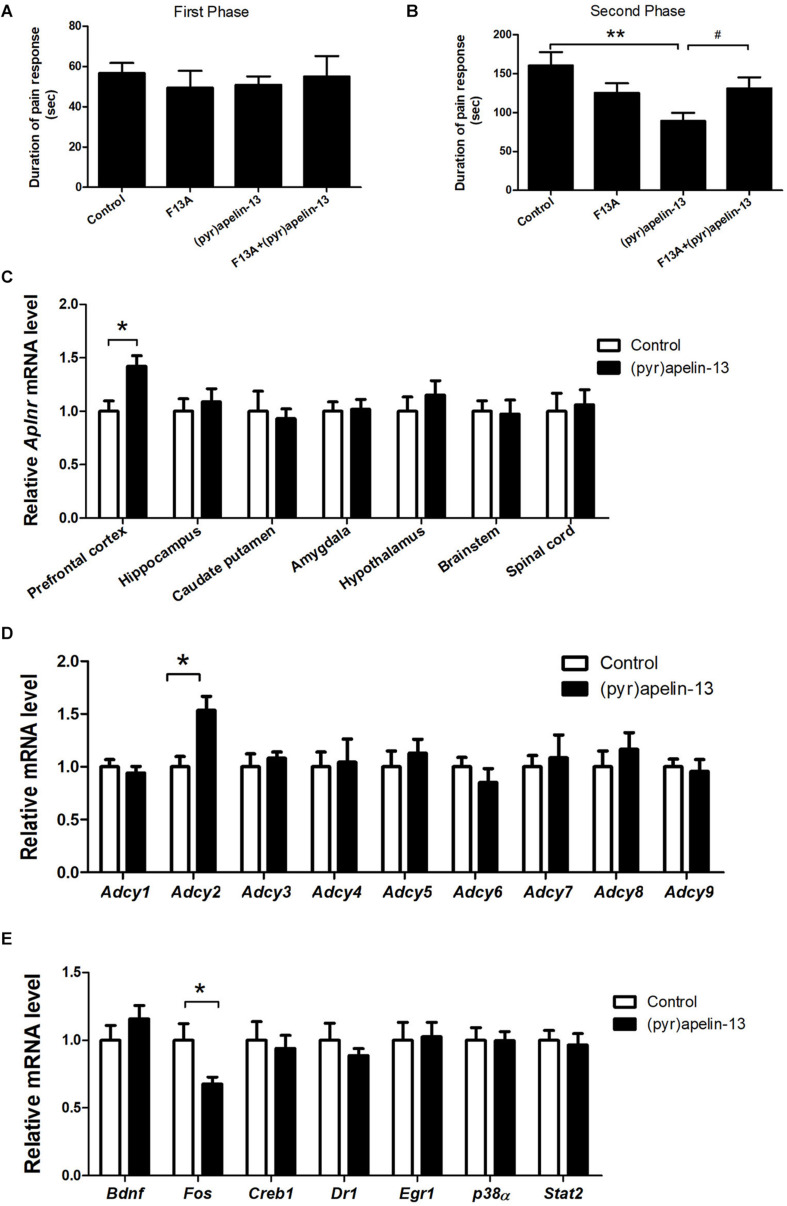
The effect of an APJ antagonist on the antinociception of (pyr)apelin-13, the influence of i.v. (pyr)apelin-13 on *APJ* gene expression in different brain regions, as well as *Adcy1*–*Adcy9* gene expression in the prefrontal cortex. **(A,B)** The effect of an APJ antagonist on apelin-13(F13A) (20 mg/kg, i.v.) and the antinociception of (pyr)apelin-13 (20 mg/kg, i.v.) in the formalin test. The apelin-13(F13A) was i.v. co-administrated with (pyr)apelin-13. The data were analyzed by ANOVA followed by Dunnett’s test. **(C)** The effect of (pyr)apelin-13 (20 mg/kg, i.v.) on *APJ* mRNA levels in the prefrontal cortex, hippocampus, caudate putamen, amygdala, hypothalamus, brainstem, and spinal cord. **(D)** The effect of (pyr)apelin-13 on *Adcy1*, *Adcy2*, *Adcy3*, *Adcy4*, *Adcy5*, *Adcy6*, *Adcy7*, *Adcy8*, and *Adcy9* mRNA levels in the mouse prefrontal cortex. **(E)** The effect of (pyr)apelin-13 on *Bdnf*, *Fos*, *Creb1*, *Dr1*, *Egr1*, *p38α*, and *Stat2* mRNA level in the mouse prefrontal cortex. The unpaired t-test was performed to test the difference between (pyr)apelin and the control group **(C–E)**. Data are expressed as means ± SEM. *n* = 8–10 per group. *p < 0.05, **p < 0.01 vs. control; ^#^*p* < 0.05 vs. (pyr)apelin-13 treated group.

To determine whether the APJ receptor mediated the antinociception effect of i.v. (pyr)apelin-13, the APJ receptor antagonist, apelin-13(F13A), was administered and *Aplnr* mRNA was assessed in different mouse brain regions. Apelin-13(F13A) (20 mg/kg) alone had no influence on licking/biting time in either phase 1 (*p* = 0.442, Figure 2A) or phase 2 (*p* = 0.137, Figure 2B) during the formalin test. However, co-administration (i.v.) of apelin-13(F13A) (20 mg/kg) with (pyr)apelin-13 (20 mg/kg) significantly blocked the antinociceptive effect of (pyr)apelin-13 during phase 2 (*p* < 0.05, Figure 2B). Moreover, i.v. administration of (pyr)apelin-13 significantly upregulated *Aplnr* gene expression only in the mouse prefrontal cortex (*p* < 0.01), but not in the hippocampus (*p* = 0.613), caudate putamen (*p* = 0.742), amygdala (*p* = 0.898), hypothalamus (*p* = 0.435), brainstem (*p* = 0.869), or spinal cord (*p* = 0.783), compared with the control (Figure 2C).

### The Effect of i.v. Administration of (Pyr)Apelin-13 on Gene Expression

To explore the potential signals involved in the antinociception of (pyr)apelin-13, the G protein-coupled receptor APJ’s downstream genes *Adcy1*–*Adcy9*, *Bdnf*, *Fos*, *Creb1*, and others were assessed in the prefrontal cortex. (pyr)Apelin-13 significantly upregulated gene expression of *Adcy2* (*p* < 0.05), but not the other *Adcy*s (Figure 2D). (pyr)Apelin-13 obviously downregulated *Fos* gene expression (*p* < 0.05). However, (pyr)apelin-13 had no influence on the expression of *Bdnf*, *Creb1*, *Dr1*, *Egr1*, *p38α*, or *Stat2* (Figure 2E).

### The Effect of Naloxone and Nor-BNI on the Antinociception Effect of i.v. Administered (Pyr)Apelin-13

**FIGURE 3:**
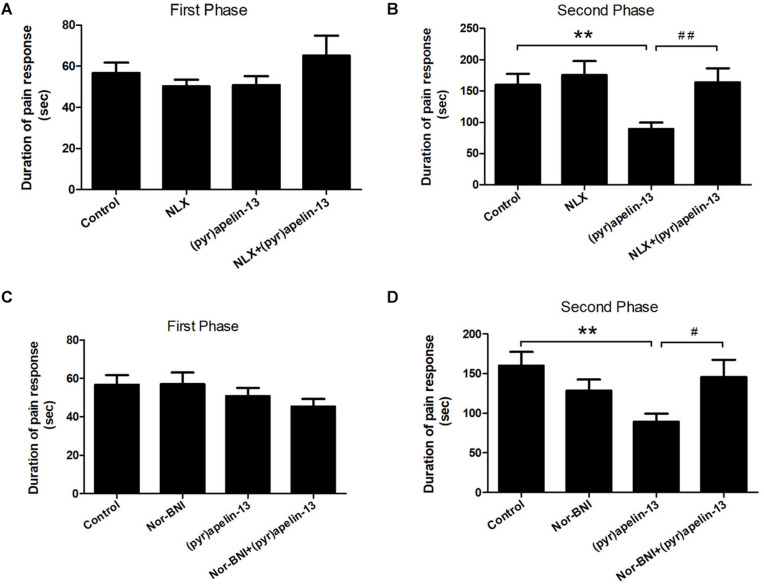
The effect of opioid receptor antagonists on the antinociception of (pyr)apelin-13. **(A,B)** The influence of non-selective opioid receptor antagonist naloxone (NLX, 2 mg/kg) on the antinociceptive effect induced by (pyr)apelin-13 (20 mg/kg) in the formalin test. **(C,D)** The influence of selective KOR antagonist, nor-Binaltorphimine (nor-BNI, 10 mg/kg), on the antinociceptive effect induced by (pyr)apelin-13 in the formalin test. The NLX or nor-BNI was i.v. co-administrated with (pyr)apelin-13, respectively. Values are presented as means ± SEM. *n* = 7–10/group. **p < 0.01 vs. control; ^#^*p* < 0.05 and ^##^*p* < 0.01 vs. (pyr)apelin-13 treated group.

To further investigate the involvement of the opioid receptors in the antinociceptive effect of (pyr)apelin-13, the non-specific opioid receptor antagonist naloxone and the specific KOR antagonist nor-BNI were evaluated. Naloxone (2 mg/kg) had no effect on licking/biting during the first (*p* = 0.312) or the second phase (*p* = 0.591) of the formalin test, when compared with the control (Figures 3A,B). However, i.v. co-administration of naloxone markedly reversed the antinociceptive response induced by (pyr)apelin-13 (20 mg/kg) during the second phase of the formalin test (*p* < 0.01, Figure 3B). As shown in Figures 3C,D, compared with saline treatment, nor-BNI (10 mg/kg) had no influence on the formalin-induced nociceptive behavior during the first (*p* = 0.980) or the second phase (*p* = 0.188). However, co-administration of nor-BNI significantly antagonized the analgesic effect of (pyr)apelin-13 (20 mg/kg) during the second phase of the formalin test (*p* < 0.01, Figure 3D).

### The Effect of (Pyr)Apelin-13 on Gene Expression and Levels of Endogenous Opioid Peptides and Opioid Receptors

**FIGURE 4:**
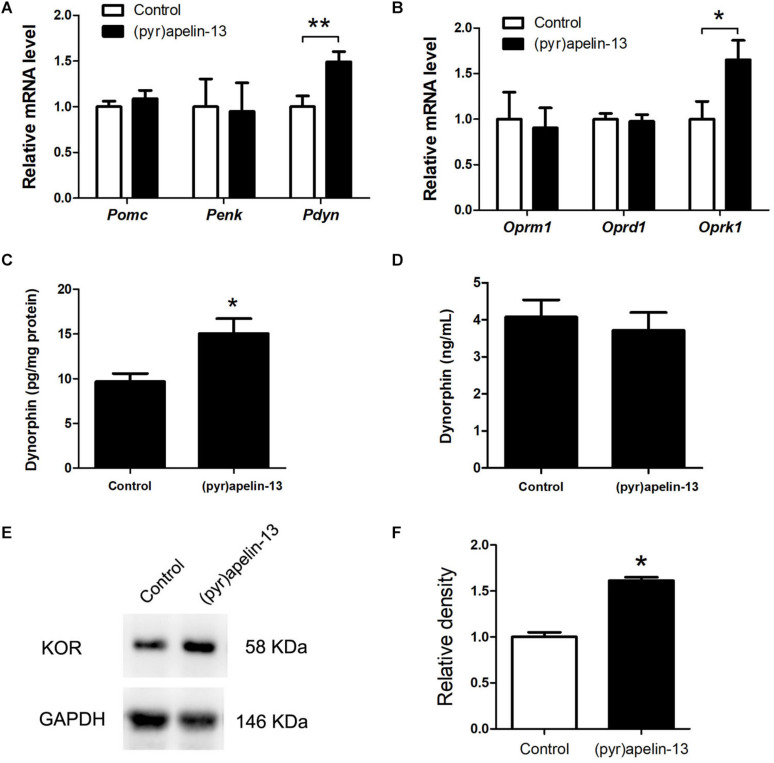
The effect of (pyr)apelin-13 on endogenous opioid peptides, opioid receptor gene and protein expression, and the content of dynorphin in the prefrontal cortex or serum in mice. **(A,B)** The effect of (pyr)apelin-13 (20 mg/kg, i.v.) on the relative mRNA levels of *Pomc*, *Penk*, *Pdyn*, *Oprm1*, *Oprd1*, and *Oprk1* normalized to the housekeeping gene *36B4* mRNA levels by real-time PCR. The concentration of dynorphin in the prefrontal cortex **(C)** and serum **(D)** was assessed by ELISA. Representative western blot **(E)** and relative protein levels **(F)** of the KOR, normalized to the housekeeping protein GAPDH. Data are presented as means ± SEM. The difference between (pyr)apelin-13 and the control group was analyzed by the unpaired *t*-test. *n* = 5–8/group. *p < 0.05 and **p < 0.01 vs. control.

To determine which endogenous opioid peptides were involved in the antinociception effect of (pyr)apelin-13, the mRNAs level of *Pomc*, *Penk*, and *Pdyn* in the prefrontal cortex were determined. As shown in Figure 4A, i.v. administration of (pyr)apelin-13 did not influence *Pomc* (*p* = 0.437) or *Penk* (*p* = 0.912) expression compared with saline treatment. However, *Pdyn* expression was significantly upregulated (*p* < 0.05). To identify which type of opioid receptor participated in the analgesic effect of (pyr)apelin-13, the expression levels of *Oprm1*, *Oprd1*, and *Oprk1* were analyzed. Neither *Oprm1* (*p* = 0.796) nor *Oprd1* (*p* = 0.808) expression levels were changed by (pyr)apelin-13, compared with the control (Figure 4B). In contrast, *Oprk1* gene expression was obviously increased (*p* < 0.05). ELISA found that (pyr)apelin-13 significantly upregulated the levels of dynorphin in the prefrontal cortex (*p* < 0.05, Figure 4C), but not in the serum, when compared with the control (*p* = 0.596, Figure 4D). Western blot analysis demonstrated the protein level of the KOR to be significantly increased in the prefrontal cortex in animals treated with (pyr)apelin-13-treated, compared with the control (*p* < 0.05, Figures 4E,F).

**FIGURE 5:**
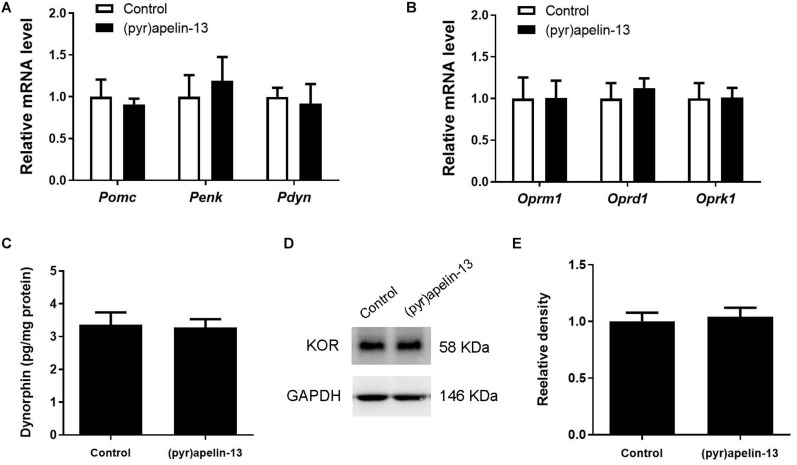
The influence of (pyr)apelin-13 on endogenous opioid peptides, opioid receptor gene and protein expression, and the content of dynorphin in the striatum in mice. **(A,B)** The effect of (pyr)apelin-13 (20 mg/kg, i.v.) on the relative mRNA levels of *Pomc*, *Penk*, *Pdyn*, *Oprm1*, *Oprd1*, and *Oprk1* in striatum. They were normalized to the housekeeping gene *36B4* mRNA levels by real-time PCR. The concentration of dynorphin in the striatum **(C)** was assessed by ELISA. Representative western blot **(D)** and relative protein levels **(E)** of the KOR, normalized to the housekeeping protein GAPDH. The unpaired t-test was performed to compare the difference between the (pyr)apelin-13-treated group and control. Data are presented as means ± SEM. *n* = 6–10/group.

To explore whether (pyr)apelin-13 has an influence on dynorphin/KOR in striatum, the mRNA and protein levels of dynorphin/KOR were detected. As shown in Figures 5A,B, (pyr)apelin-13 did not produce a significant influence on *Pomc* (*p* = 0.683), *Penk* (*p* = 0.640), *Pdyn* (*p* = 758), *Oprm1* (*p* = 0.985), *Oprd1* (*p* = 0.576), or *Oprk1* (*p* = 0.962) in striatum, compared with the saline-treated group. ELISA and western blot results demonstrated that (pyr)apelin-13 did change the content of dynorphin (*p* = 0.854, Figure 5C) or KOR expression (*p* = 0.725, Figures 5D,E) in striatum, compared with the control.

### The Effect of (Pyr)Apelin-13 on Motor Function and Light/Dark Aversion

To examine whether (pyr)apelin-13 could induce side effects of motor impairment and aversion during the process of antinociception, we detect the related indexes using the open field test, wire hanging test, and light/dark aversion test. Intravenous administration of (pyr)apelin-13 (2, 10, and 20 mg/kg) had no influence on total distance traveled [*F*(3, 36) = 0.201, *p* = 0.895], average velocity [*F*(3, 36) = 0.201, *p* = 0.895], or spontaneous activity [*F*(3, 31) = 0.658, *p* = 0.583] in the open field test (Table 2). (pyr)Apelin-13 (2, 10, and 20 mg/kg) did not change the score [*F*(3, 36) = 0.326, *p* = 0.807] or latency [*F*(3, 36) = 0.186, *p* = 0.905] in the wire hanging test (Table 3). In the light/dark aversion, (pyr)apelin-13 (2, 10, and 20 mg/kg) had no effect on the number of transitions [*F*(3, 33) = 0.290, *p* = 0.832] or time (s) lit area [*F*(3, 33) = 0.792, *p* = 0.507, Table 4].

**TABLE 2:** Effect of (pyr)apelin-13 on open field test in mice.

Treatment	Dose (mg/kg)	*n*	Total distance traveled (mm)	Average velocity (mm/s)	Spontaneous activity (*n*)
Vehicle	–	11	15939.11 ± 2229.23	8.85 ± 1.24	281.18 ± 13.80
(pyr)Apelin-13	2	10	16044.83 ± 4131.48	8.91 ± 2.30	245.40 ± 21.63
(pyr)Apelin-13	10	9	12940.84 ± 1762.58	7.19 ± 0.98	259.33 ± 19.98
(pyr)Apelin-13	20	10	14557.33 ± 3818.45	8.09 ± 2.12	248.00 ± 26.40

*ANOVA was used to compare the difference between (pyr)apelin-13 and vehicle.*

**TABLE 3:** Effect of (pyr)apelin-13 on the wire hanging test in mice.

Treatment	Dose (mg/kg)	*n*	Score	Latency (s)
Vehicle	–	10	3.53 ± 0.43	11.50 ± 1.78
(pyr)Apelin-13	2	10	3.63 ± 0.39	13.33 ± 2.51
(pyr)Apelin-13	10	10	3.73 ± 0.38	12.90 ± 1.71
(pyr)Apelin-13	20	10	4.03 ± 0.30	13.57 ± 2.45

*ANOVA was used to compare the difference between (pyr)apelin-13 and vehicle.*

**TABLE 4:** Effect of (pyr)apelin-13 in the light/dark aversion test in mice.

Treatment	Dose (mg/kg)	*n*	Number of transitions/5 min	Time (s) lit area/5 min
Vehicle	–	10	8.90 ± 3.41	244.20 ± 23.85
(pyr)Apelin-13	2	9	10.44 ± 3.79	233.00 ± 22.52
(pyr)Apelin-13	10	9	12.00 ± 2.12	224.67 ± 18.25
(pyr)Apelin-13	20	9	8.00 ± 3.34	267.33 ± 15.29

*ANOVA was used to compare the difference between (pyr)apelin-13 and vehicle.*

## Discussion

This study demonstrated that i.v. or i.m. injection of (pyr)apelin-13 or apelin-13 produced an antinociceptive effect in a murine formalin-induced paw inflammatory pain model. The antinociception of i.v. (pyr)apelin-13 was significantly antagonized by antagonist for the APJ receptor, the opioid receptor, and the KOR. (pyr)Apelin-13 (i.v., 20 mg/kg) upregulated *Aplnr* and *Adcy2* gene expression and downregulated *Fos* gene expression in the prefrontal cortex of the mice. Also, (pyr)apelin-13 produced an increase in the mRNA and protein levels of dynorphin and KOR in the prefrontal cortex, not striatum. In addition, (pyr)apelin-13 did produce side effects of motor impairment and aversion during the process of antinociception.

The formalin test is a model of tonic continuous pain induced by injured tissue and is a valid model for clinical pain (Tjolsen et al., 1992). This model is typically used to evaluate the tonic analgesic effect of pharmacological agents in that the model is reproducible and provides a quantifiable behavioral response (Reeta et al., 2006). Our results demonstrated a characteristic biphasic pain response induced by 1% formalin in the mouse hind paw, similar to previous reports (Tjolsen et al., 1992; Abbadie et al., 1997). The first phase (0–10 min) is produced by direct activation of nociceptive neurons by formalin, with the second phase (10–30 min) due to an inflammatory response to tissue injury (Abbott et al., 1995). This study demonstrated (pyr)apelin-13 (10 and 20 mg/kg, i.v.) to inhibit pain behavior during the second phase, but not during the first phase of the formalin test. Apelin-13 (10 mg/kg, i.v.) produced an antinociceptive effect during the first phase, but not the second phase of the formalin test. These observations may be due to characteristics (such as stability, degradation) of the different molecular forms of apelin-13. Further, (pyr)apelin-13 (20 mg/kg, i.m.) produced an antinociceptive response during the second phase of the formalin test, not the first. These results demonstrate peripheral (pyr)apelin-13 to mitigate inflammatory pain. The discrepancy results of (pyr)apelin-13 in the first phase and the second phase of the formalin test may be due to their different modulatory mechanisms of the two phases. The first phase was caused by a bursting activity from pain fibers, such as C fibers, whereas the second phase was caused by inflammation and central sensitization (Tjolsen et al., 1992; McNamara et al., 2007).

Apelin is an endogenous ligand for APJ, which is a seven-transmembrane GPCR (Tatemoto et al., 1998). Apelin-13(F13A) is a specific antagonist of the apelin receptor and has been used to explore the mechanistic basis for the hypotensive effect induced by apelin-13 (Lee et al., 2005). Our results demonstrate apelin-13(F13A) to significantly block the antinociceptive effect of i.v. (pyr)apelin-13 during the second phase of the mouse formalin test, suggesting the involvement of APJ in the antinociception induced by i.v. administration of (pyr)apelin-13. These results are in accordance with a previous report in which APJ was shown to be involved in the antinociception of apelin-13 in a mouse visceral pain model (Lv et al., 2012).

The prefrontal cortex is important for pain processing (Ong et al., 2019). By meta-analysis of experimental pain studies, Apkarian et al. (2005) concluded that the prefrontal cortex, anterior cingulate cortex, insular cortex, and other regions of the brain are positively associated with pain. Our results indicate that i.v. administration of (pyr)apelin-13 upregulated *Aplnr* gene expression in the mouse prefrontal cortex, but not in the hippocampus, caudate putamen, amygdala, hypothalamus, brainstem, or spinal cord. Hence, the effect of peripheral (pyr)apelin-13 is on the brain, especially within the prefrontal cortex, during inflammatory pain.

Adenylate cyclase (ADCY) is APJ’s downstream signaling molecule (Burghi et al., 2019). We found that i.v. administration of (pyr)apelin-13 upregulated *Adcy2* gene expression in the prefrontal cortex of formalin-treated mice. Thus, (pyr)apelin-13 likely exerts its antinociceptive effect by activating APJ/ADCY2. In addition, we found that *Fos* gene expression was downregulated by (pyr)apelin-13 in this formalin-induced inflammatory pain model. Fos is believed to be a neural marker of pain. Fos expression most likely reflects the role that the central nervous system plays in the stress response elicited by pain (Harris, 1998). Therefore, the inhibitory effect of (pyr)apelin-13 on inflammatory pain would be followed by a decrease in the pain marker, Fos.

**FIGURE 6:**
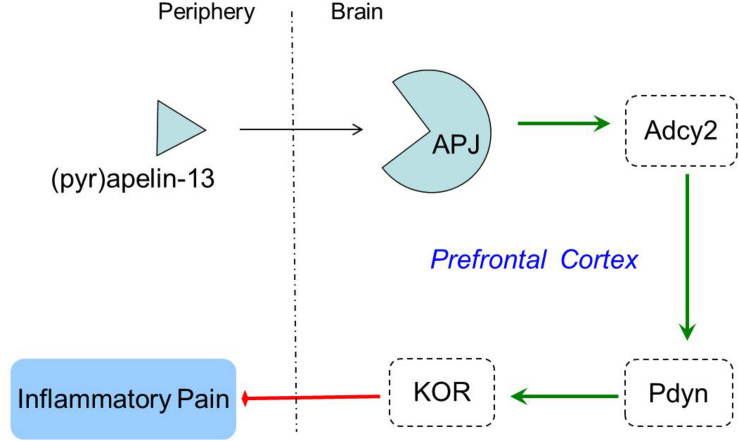
The potential mechanisms of peripheral (pyr)apelin-13 on inflammatory pain.

Opioid systems play an important role in the modulation of pain behavior and antinociception. Our study demonstrated that the non-selective opioid receptor antagonist, naloxone, blocked the antinociception of (pyr)apelin-13 during the second phase of the formalin test, suggesting the involvement of the opioid receptor in the inhibitory effect of (pyr)apelin-13 on inflammatory pain. The classified receptor subtypes include mu (μ), kappa (κ), and delta (δ) opioid receptors. The corresponding precursors of these endogenous opioid peptides are proopiomelanocortin (POMC), prodynorphin (PDYN), and preproenkephalin (PENK). Recent studies indicated that APJ formed a heterodimer with KOR, suggesting a close relationship between apelin/APJ system and KOR (Li et al., 2012; Rostamzadeh et al., 2017). We found i.v. (pyr)apelin-13 to significantly upregulate *Pdyn* and *Oprk1* gene expression in the mouse prefrontal cortex, but not *Pomc*, *Penk*, *Oprm1*, or *Oprd1*. Moreover, we found dynorphin and KOR levels in the prefrontal cortex to be significantly increased as judged by ELISA and western blot analysis. Intriguingly, similar variations in transcript and protein levels of PDYN and KOR indicated that the antinociception of (pyr)apelin-13 may be mediated by the PDYN/KOR system. In addition, the KOR antagonist, nor-BNI, antagonized the antinociceptive effect induced by (pyr)apelin-13 during the second phase of the formalin test, which confirms the involvement of KOR. This result is supported by previous reports that the release of dynorphin plays a role in the control of inflammatory pain (Cabot et al., 2001) and that KOR is involved in regulation of nociceptive behaviors in a variety of animal pain models (Kivell and Prisinzano, 2010). Striatum played an important role in pain modulation (Barcelo et al., 2012). Unfortunately, we found that (pyr)apelin-13 did not influence PDYN/KOR gene or protein expression in striatum. Based on these results, we speculated that i.v. injection of (pyr)apelin-13 induced an increase of (pyr)apelin-13 in brain, activated the APJ in prefrontal cortex first, stimulated *Adcy2* gene expression, and then exerted the dynorphin/KOR, thereby exhibiting an antinociceptive effect against inflammatory pain (as shown in Figure 6). Some analgesics were commonly accompanied by side effects (Meymandi et al., 2006). Our present study demonstrated that (pyr)apelin-13 did not influence motor function and light/dark aversion, suggesting a potential analgesic compound for (pyr)apelin-13.

## Conclusion

In summary, the peripheral administration of (pyr)apelin-13 produced an antinociceptive effect during the inflammatory phase (second phase) of the mouse formalin test. The antinociception of (pyr)apelin-13 was mediated through the APJ receptor, which activated the endogenous dynorphin/KOR system in the prefrontal cortex of mice. This study may pave the way for a new strategy for the investigation and control of inflammatory pain.

## Data Availability Statement

The raw data supporting the conclusions of this article will be made available by the authors, without undue reservation, to any qualified researcher.

## Ethics Statement

The animal study was reviewed and approved by the Committee of Medical Ethics and Welfare for Experimental Animals, Henan University School of Medicine.

## Author Contributions

YY and XW developed the idea, designed the research, and contributed to revise the writing. SL, XZ, YF, YZ, and BC performed the experiment and analyzed the data. SL wrote the draft of the article. All authors contributed to the article and approved the submitted version.

## Conflict of Interest

The authors declare that the research was conducted in the absence of any commercial or financial relationships that could be construed as a potential conflict of interest.
